# Medical Tourism: A Cost or Benefit to the NHS?

**DOI:** 10.1371/journal.pone.0070406

**Published:** 2013-10-24

**Authors:** Johanna Hanefeld, Daniel Horsfall, Neil Lunt, Richard Smith

**Affiliations:** 1 Department Global Health and Development, Faculty of Public Health and Policy, London School of Hygiene and Tropical Medicine, London, United Kingdom; 2 Department of Social Work and Social Policy, University of York, York, United Kingdom; 3 Faculty of Public Health & Policy, London School of Hygiene & Tropical Medicine, London, United Kingdom; Erasmus University Rotterdam, The Netherlands

## Abstract

‘Medical Tourism’ – the phenomenon of people travelling abroad to access medical treatment - has received increasing attention in academic and popular media. This paper reports findings from a study examining effect of inbound and outbound medical tourism on the UK NHS, by estimating volume of medical tourism and associated costs and benefits. A mixed methods study it includes analysis of the UK International Passenger Survey (IPS); interviews with 77 returning UK medical tourists, 63 policymakers, NHS managers and medical tourism industry actors policymakers, and a review of published literature. These informed costing of three types of treatments for which patients commonly travel abroad: fertility treatment, cosmetic and bariatric surgery. Costing of inbound tourism relied on data obtained through 28 Freedom-of-Information requests to NHS Foundation Trusts. Findings demonstrate that contrary to some popular media reports, far from being a net importer of patients, the UK is now a clear net exporter of medical travellers. In 2010, an estimated 63,000 UK residents travelled for treatment, while around 52,000 patients sought treatment in the UK. Inbound medical tourists treated as private patients within NHS facilities may be especially profitable when compared to UK private patients, yielding close to a quarter of revenue from only 7% of volume in the data examined. Costs arise where patients travel abroad and return with complications. Analysis also indicates possible savings especially in future health care and social costs averted. These are likely to be specific to procedures and conditions treated. UK medical tourism is a growing phenomenon that presents risks and opportunities to the NHS. To fully understand its implications and guide policy on issues such as NHS global activities and patient safety will require investment in further research and monitoring. Results point to likely impact of medical tourism in other universal public health systems.

## Introduction

The phenomenon of people travelling abroad to access medical treatment – commonly termed ‘Medical Tourism’ – has received increasing attention in academic and popular media [1]. The confluence of available and affordable air travel, internet-based marketing by providers, and an increasing requirement for out-of-pocket expenditure, even in universal public health care systems such as the UK NHS, suggests that increasing numbers of patients may consider travelling for treatment. The PIP scandal highlighted challenges for UK patients in seeking redress from private providers, especially where these may be based in other jurisdictions [2], [3].

As the new NHS reforms introduce yet greater market elements, including the removal of the cap on income from private patients [4], and the EU Directive on crossborder healthcare is implemented which codifies rights around patient mobility [5], it is imperative to consider the challenges and opportunities that medical tourism – inward and outward – may present to the NHS [6].

Yet, reliable information on even the basic number, characteristics, motivations and experiences of such patients is scarce, as patients arrange and pay for such care privately [7]. Indeed, a recent review of medical tourism literature [8] found that academic literature relies heavily on opaque data from private consultancy firms or unverified media reports [9], [10]. In the absence of even the basic level of information in these areas, it is understandable that rhetoric has filled the vacuum. In this paper we present evidence from the largest study yet conducted concerning medical tourism, undertaken from an NHS perspective, to provide a firmer footing for debate and discussion by health professionals, NHS managers and those involved in the wider policy-making context.

## Methods

Authors interviewed 77 UK medical tourists and 63 other UK stakeholders between March 2011 and August 2012. Interviewees gave written consent to participate in the study. Interviews were recorded, transcribed and thematically analysed. The study received ethical clearance from the National NHS Ethics review process submitted through the Sheffield Research Ethics Committee approval (11/H1308/3).

Analysis is three-fold: (i) the volume and characteristics of outbound and inbound UK medical tourists is based upon the International Passenger Survey (IPS); (ii) assessment of NHS income from foreign patients is based upon freedom-of-information requests submitted to 28 NHS Foundation Trust hospitals; and (iii) evaluation of the challenges encountered, costs incurred and potential savings for the NHS is based on a review of published and grey literature and interviews with UK nationals, NHS managers and policy makers. Each of these is described below.

### Analysis of the International Passenger Survey (IPS)

The IPS, conducted by the UK Office of National Statistics (ONS), collects information from passengers as they enter or leave the UK. Passengers are randomly selected as they travel through passport control and a brief survey is administered. One of the survey questions asks passengers to define their primary purpose for travel; ‘medical treatment’ is one of the answers recorded, thus providing insight into the number of passengers who self-declare that they are travelling for medical treatment.

The IPS dataset from 2000–2010, from the Office of National Statistics (ONS), was analysed by two authors independently, triangulating results. Data from the IPS, interviews, literature and NHS tariffs were used to calculate cost impacts. Authors used the different data sources accessed to carefully triangulate and better understand the reliability of the data from the IPS, which is reflected on in the discussion.

### FOI Requests

Submitted to 28 Foundation Trust hospitals on volume and income from international private patients. Trusts were purposely selected to be those most likely to be visited by inbound tourists i.e., large and well-known Trusts, such as Great Ormond Street Hospital for Sick Children, many of which are based in London. Data on foreign patients was analysed to understand the potential of earnings from foreign patients.

### Qualitative Analysis

Authors interviewed 77 UK residents who travelled abroad for treatment and 63 other UK stakeholders between March 2011 and August 2012. Interviews were recorded, transcribed and thematically analysed. The study received ethical clearance from the National NHS Ethics review process.

## Results

**Figure 1:**
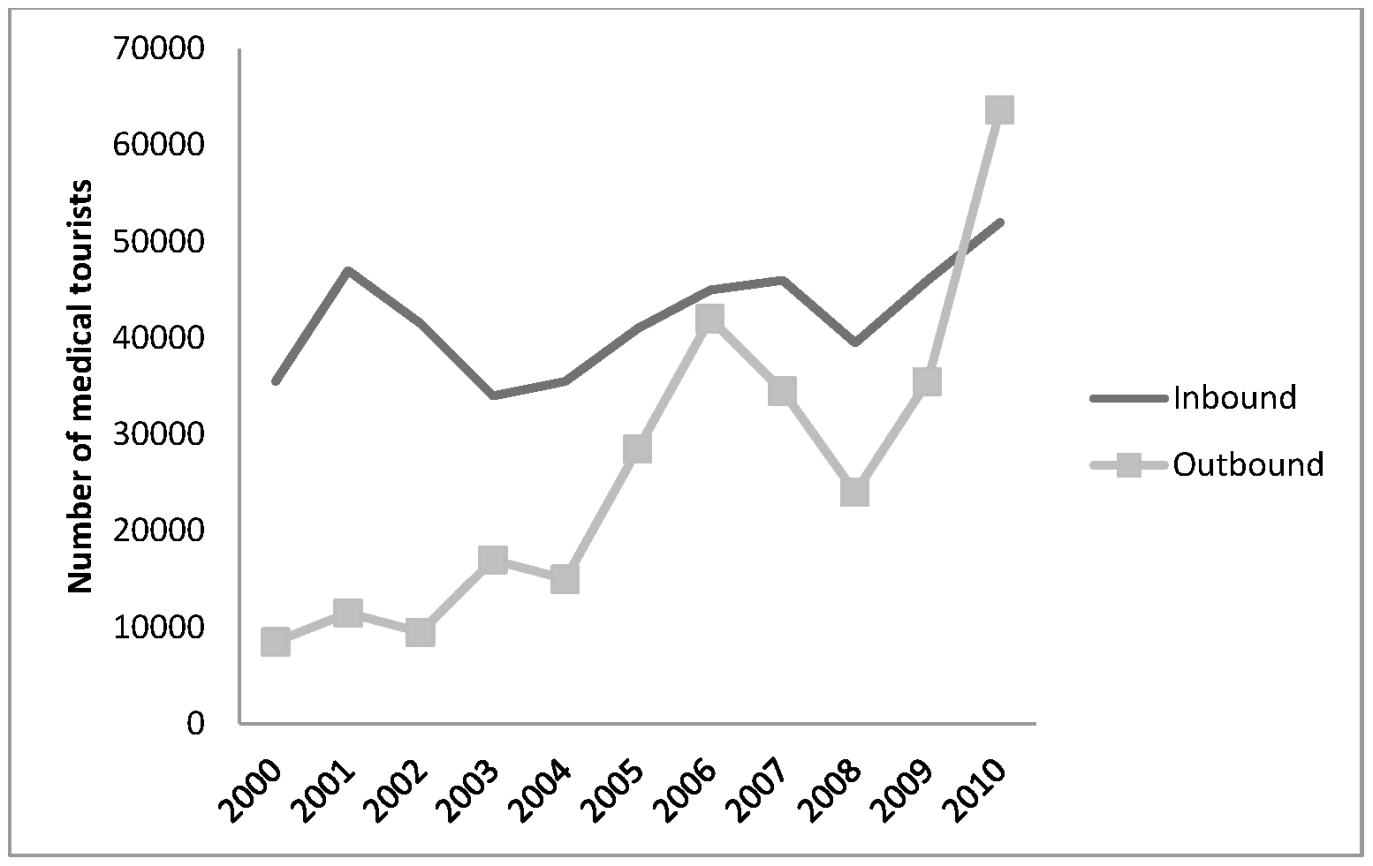
The number of people who travelled into or out of the UK for medical treatment during the period 2000–2010.

While the level of inward travel of foreign patients to the UK (although not necessarily the NHS) has been relatively stable over the last decade, there has been a substantial increase in the number of UK residents travelling abroad to access medical treatment, as indicated in Figure 1.

### Destination of UK Outbound Medical Travellers

**Figure 2:**
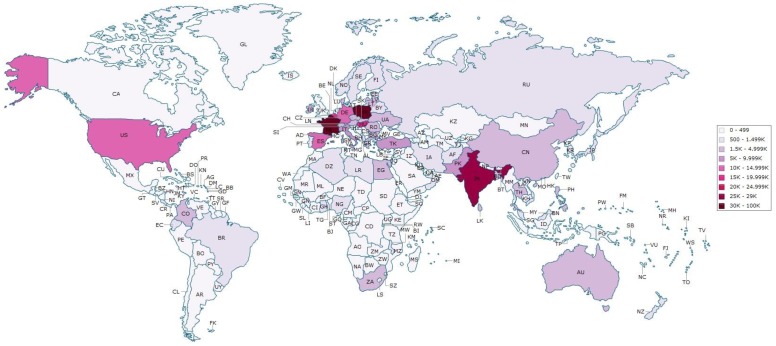
Map depicting total numbers of medical travellers and their destinations from the UK over the period 2000–2010.

Figure 2 shows UK residents most commonly travel for medical treatment to North, West, and Southern Europe with France being the most visited country over the decade.

**Figure 3:**
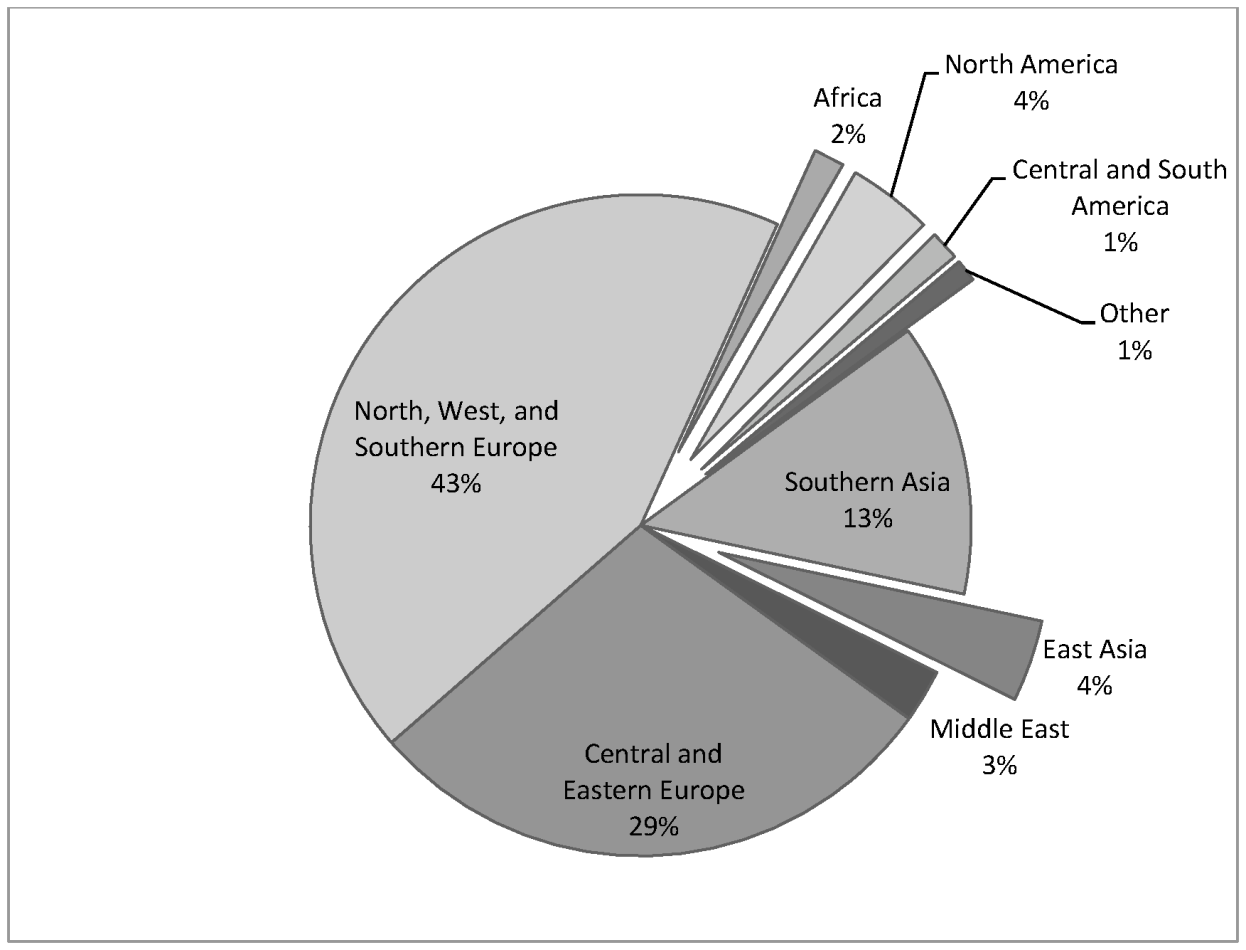
Pie Chart showing total outward medical travel by UK residents by destination region over the time-period 2000–2010.

Examining this in greater detail (Fig 3) suggests that Central and Eastern Europe are second most popular, and that Poland and Hungary are increasingly popular.

South Asia, primarily India, also attracts large numbers of UK patients, making it the most frequently visited non-European country, with a relatively stable pattern of travel to India, Pakistan, and in much lower numbers Sri Lanka and Bangladesh, possibly reflecting a diaspora effect. In contrast, East Asia shows a different pattern, with virtually no medical travellers recorded by the IPS prior to 2003, yet by 2010 15% of all UK medical travellers went to East Asia. This increase of 430% is unlikely to be solely related to diaspora patients, but does correlate with many South East Asian countries marketing strategies at this time [11].

Based on the IPS data, and patient interviews, treatment specific destinations emerge. For example, UK dental patients increasingly travel to Hungary and Poland, which corresponds to the varied availability of NHS dental treatment over the last decade [12]. Fertility tourists often travel to countries in Eastern Europe, Cyprus and Spain possibly owing to more easily accessible gametes, and less stringent regulation which allows anonymous donation as well as a greater number of embryos transferred [13].

### Inward Medical Travel

As evident in Figure 1, data from the IPS suggests that international patient inflows to the UK (independent sector and NHS private services) were in the region of 52,000 in 2010. Data over the decade also confirms that while growing, the overall numbers of patients travelling into the UK to access medical services is rising at a much slower rate than UK residents travelling out for care. So, contrary to some popular media reports, far from being a net importer of patients, the UK is now a clear net exporter of medical travellers.

**Figure 4:**
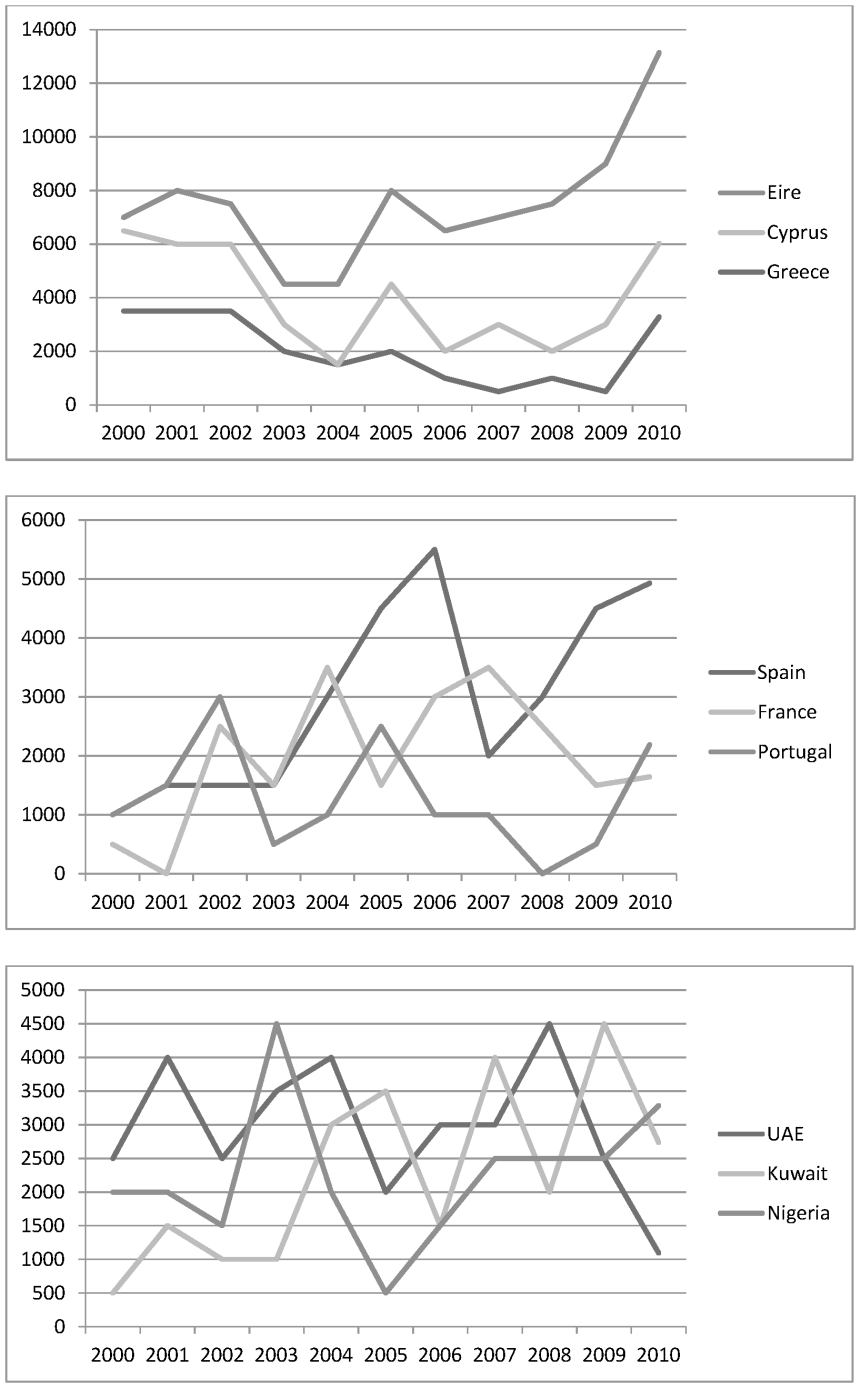
Nine most common countries of origin for those who travelled to the UK for medical (2000–2010).

Major source countries for patients coming into the UK include Spain, Greece, Cyprus and the Middle East. The number of Greeks and Cypriots travelling into the UK to access treatment rose rapidly in 2009 and 2010. These figures may reflect a change as a result of the economic crisis, which in turn has meant severe public sector cuts in these countries, including in health [14]. Similarly, while medical tourists from Ireland may choose to travel to access treatment not available there, including termination of pregnancies, the rapid increase in patients from Ireland in recent years may reflect the cuts in the health sector there and greater numbers of UK citizens resident in Ireland returning to the UK for treatments (see Figure 4). The ‘dip’ in both inbound and outbound medical travel evident in Figure 1 in 2008 may be attributable to the onset of the crisis. Examining the number of travellers by quarter found a much lower number of inward and outward medical travellers in Quarter 3 of 2008 during the onset of the crisis, than the rest of the year, or Quarter 3 in 2009. In the case of Irish, Spanish (and perhaps French) residents, it is highly likely that a substantial number will be UK expats and it is unclear whether these engage in out-of-pocket medical treatment (in the private sector or NHS) or whether they accept NHS services free at the point of use for which they may (or may not) be eligible.

A further significant number of patients travel from the Middle East (specifically from the United Arab Emirates and Kuwait) although visitor numbers from both countries dropped sharply in 2008 and 2009 respectively. Despite some variation between years, a stable inward flow of medical travellers from Nigeria is also evident over the past decade, perhaps reflecting the growing wealth of some sections of that population.

#### International activity within hospital trusts

Our Freedom-of-Information requests suggest that Trusts could not always clearly identify international patients within their pool of private patients because nationality was not recorded when they underwent treatment and nationality/place of residence may differ. Looking at the 28 Trusts within our sample, their international activity ranged from relatively marginal to being one-third of their total private work.

Where Trust managers were interviewed (at seven sites) they spoke of international patient flows and activities within the context of pressure on NHS resources, and pre-existing international activities and linkages. Commercial imperatives were balanced with strong statements regarding the core NHS role, centred on NHS services and prioritising NHS patient care. International patient activity was typically specialist where it was not possible to treat locally because of relatively small volumes and the complex nature of treatment required. Relationships, primarily clinical ones, for example where a clinician from aboard had trained or worked in a UK hospital were paramount in maintaining flows of international patients. Established practices of education, training, consultancy and linkages were reported to help facilitate referrals. Rather than systematic links these personal networks appeared paramount in linking UK hospitals to international patients.

### What is the Impact of Medical Tourism on the NHS ‘Bottom-line’?

Using the IPS data, analysis from interviews with medical tourists, academic literature and published NHS data we calculated possible costs and savings for the NHS for three types of medical tourism identified (see annex S1 for calculations).

#### Fertility tourism

Based on data from the Office of National Statistics on multiple births in the UK and evidence from a hospital in London which found over a quarter of multiple births were in women who had travelled abroad for fertility treatment [15], we estimated the cost incurred through multiple births as a result of individuals travelling abroad for fertility treatment. Multiple pregnancies pose risks to mothers and children. We concentrated on the actual costs of multiple births per se as the exact needs throughout pregnancy and possible complications are highly variable between women, and thus our estimates will be highly conservative. We calculated the additional cost of a twin or triplet over singleton birth resulting from fertility travel in 2010 to be £15.5 million.

The long-term costs resulting from assisted reproductive technologies, including multiple pregnancies will not differ between medical tourists and fertility patients who received care in the UK. However, our research indicates that patients will travel in search of reproductive care to countries with regulations that will allow fertility treatment likely to result in a higher number of multiple births. Any effort to address the rise in multiple births in the UK therefore needs to take account of medical travel and involve specific targeted information to be effective.

#### Cosmetic tourism

We also calculated the likely cost of complications resulting from cosmetic tourism based on a recent study by Miyagi et al. [16], who described a cohort of patients in a tertiary facility which reported problems arising from cosmetic surgery undertaken abroad over a period of three years. The authors calculated the cost of treatment provided within the NHS for complications and highlighted the reimbursement received by the hospital from the PCT (which was less than the expenditure of the hospital). Based on our calculations complications of medical tourists are at a cost of £8.2 million per annum within the NHS.

#### Bariatric surgery

Compared to other types of tourism discussed, bariatric tourism may represent savings rather than costs for the NHS, as well as wider social savings. With 25% of the UK population classified as clinically obese, the financial impact of obesity on the NHS is calculated as £4.3billion by the DoH [17]. Obesity also has wider costs for social services. For example, a study by the National Office of Accounting estimated that 18 million working days were lost due to obesity with surgery offering potential savings. Hawkins et al. [18] demonstrated that there was a 32% increase in bariatric patients in paid work after surgery.

Based on these estimates, the 13 bariatric tourists interviewed for this research would represent a saving of £112,506 (in cost of procedure and in future health care and social services savings). Even as a high estimate, the key point remains that patients travelling abroad to receive bariatric surgery are likely to represent a saving to the NHS and social services. Further research on the longitudinal effects of bariatric surgery is needed and now underway in the University of Glasgow at the Surgical Obesity Treatment Study (ScOTS).

### Income Generated by Inbound Medical Travellers

Income generated by inbound medical travellers can be divided into additional tourism revenue, capturing the general expenditure related to patients visit to the UK, and medical expenditure (revenue to hospital).

#### Tourism revenue from all inbound medical travelers

Tourism revenue by medical travellers to the UK per annum is based on the most recent IPS data for inbound medical travellers (2010). As respondents in the IPS survey specifically state they are visiting for health care, it is assumed they would not have otherwise have visited the UK, and thus are an addition to visitor/tourist numbers to the UK. Hence, any spending would be seen to be a net benefit not otherwise coming to the UK.

Based on hospital data for patients treated within NHS hospitals, it can be assumed that 20% of inbound medical travellers receive treatment as inpatients, the remainder as day-case procedures. Expenditure was calculated for patients staying in the UK for a number of different scenarios, ranging from those who stay for four days to receive outpatient treatment to patients who receive in-patient treatment for 10 days and stay a further two weeks for follow-up (see Table 1). Assumptions were based on interview data collected and on an average hospital stay of inpatients (not just medical tourists) in 2010 to 2011 from the NHS Hospital Episode Statistics. These assumptions were that: patients likely arrive some days before treatment and remain additional days to fully recuperate or even take the opportunity for additional tourism activities; people travel with one companion, and travellers from the Middle East travel with two, and that these are not captured by the IPS (based on interview data and corroborated by a 2008 national survey conducted by *Which?*). This seems reasonable given the higher foreign patient number captured from the FOI letters and interview data from patients who often reported reluctance to be identified as medical tourists possibly due to the negative public image of medical tourism, making it unlikely that accompanying persons will identify as medical tourists. Cost of accommodation was calculated at £80 per night and £100 per day as spending for patients when they were not in hospital and for their travel companions.

**Table 1:** Calculation of additional spend by incoming medical tourists and their travel companions.

Inbound medical travellers	No	Nights in hotel	Cost hotel	Expenditure	Total expenditure
	52000				
Inpatients*	10400				
Hospital for ten days 75% (75% from ME)	7800	14	8,736,000	10,920,000	19,656,000
Hospital for five days 25% (5% from ME)	2600	7	1,456,000	1,820,000	3,276,000
**Subtotal inpatients**			10,192,000	12,740,000	22,932,000
Accompanying persons inpatients					
Hospital for ten days 75% (75% from ME)	13650	24	26,208,000	32,760,000	58,968,000
Hospital for five days 25% (5% from ME)	2730	12	2,620,800	3,276,000	5,896,800
**Subtotal accompanying persons**			28,828,800	36,036,000	64,864,800
**Total inpatient and accompanying**			39,020,800	48,776,000	**87,796,800**
**Outpatients**	41600				
4 day stay (25%)	10400	4	3,328,000	4,160,000	7,488,000
7 day stay (40) (2.75% ME)	16640	7	9,318,400	11,648,000	20,966,400
14 day stay (35%) (2%ME)	14560	14	16,307,200	20,384,000	36,691,200
**Subtotal outpatients**			28,953,600	36,192,000	**65,145,600**
**Accompanying persons outpatients**	41600				
4 day stay (25%)	10400	4	3,328,000	4,160,000	7,488,000
7 day stay (40) (2.75% ME)	17098	7	9,574,880	11,968,600	21,543,480
14 day stay (35%) (2%ME)	14809	14	16,586,080	20,732,600	37,318,680
**Subtotal accompanying persons OP**	42307		29,488,960	36,861,200	**66,350,160**
**Total outpatient and accompanying**					**131,495,760**
**Total**					**219,292,560**

Calculations are summarized in Table 1 and further explained in Annex S1 suggest that, even without taking the cost of the actual medical treatment into account, medical tourists to the UK contribute around £219 million in additional ‘tourism spending’ to the UK economy per year.

#### Healthcare revenue from all inbound medical tourists

To estimate the spend on medical procedure by inbound medical tourists in NHS facilities as accurately as possible, we submitted Freedom-of-Information requests for data on income from private patients in NHS hospitals, including UK and non-UK patients, to 28 NHS Foundation hospitals. Of 28 hospitals 19 were able to provide data on the percentage of income that resulted from non-UK resident patients and number of non UK residents treated as private patients. Authors excluded Moorfields Eye Hospital, as a review of the data across different hospitals indicated this as an outlier. Given the focus on eye medicine, it has a very large number of patients visiting for outpatient procedures at a lower per cost treatments compared to other elective procedures. The remaining 18 reported a combined income from private patients of £195 million over a period of 12 months between 2010–2011.

Those who were able to provide differentiated data indicated that £42 million of their total income was from non-UK resident patients; looking across these 18 hospitals, this meant close to 25% of their private income was from incoming medical tourists. While our sample of hospitals was weighted towards large London-based facilities which do experience a higher number of international patients, income ranged vastly between hospitals surveyed: from over £20million to just £2,466 with a mean of £2.5million.

Those hospitals that were able to report numbers of patients reported a total of 6,722 patients from abroad out of a total of 88,775 private patients counted, i.e. seven percent of private patients were inbound medical tourists. It might therefore appear that medical tourists may be especially profitable, yielding close to a quarter of revenue from only 7% of volume. For a detailed listing of patients and income per hospital, see Annex S2.

## Discussion

Results confirm that a small but increasing number of UK patients are travelling abroad to receive medical treatment. Medical travel is complex and not a uniform phenomenon. The majority of UK patients travel within Europe, but an increasing number are seeking treatment further afield. Patients are traveling specifically to Poland and Hungary, and increasingly to India and East Asia. Diaspora, country-specific marketing campaigns, and specific specialism’s seem to determine patterns of flows of UK patients seeking care abroad. Patients returning from treatment abroad experience complications.

The analysis demonstrates both the possibility of costs and savings to the NHS as a result of patients travelling abroad, which need to be considered. Unsurprisingly, the largest numbers of inbound medical tourists were in the large hospitals which are internationally known for their specialism; foremost amongst these Great Ormond Street Hospital for Sick Children which reported income of over £20million from 656 patients. Data received and summarised in Appendix 2 also highlights the variation in the percentage of income that international revenue represents for hospitals; to some, especially the large hospitals in London, it marks a significant proportion of private patient income while for others it contributes a very small percentage of funding.

Our analysis of data suggests that the UK is now a net exporter of medical tourists. While incoming medical tourists may be less likely to declare treatment as primary purpose for their visit to the UK than outbound tourists, data over time clearly shows a greater acceleration in outbound over inbound medical tourists. Despite the variations in numbers of patients visiting different hospitals and in the income per patient, the number of medical tourists was comparatively smaller than the percentage of income generated by them (7% of patients generating close to 25% of private income). These figures suggest that non-UK residents travelling to the UK for medical treatment seek high-end specialist expensive procedures, and may generate substantial revenue. Additional numbers of patients for specialist procedures may also help NHS doctors with surgical learning curves.

The changing destinations of UK travellers and the differing origins of those travelling to the UK show that medical travel is a dynamic phenomenon, which can rapidly increase and change. This highlights the importance of continuous routine monitoring to understand medical tourism and to enable researchers, professionals and policymakers to better consider the costs and benefits of medical tourism to the UK.

UK residents who had travelled abroad reported experiencing complications following their return, which echoed case reports in the literature. While we calculated potential costs of these to the NHS, complications experienced also pose an ethical question. There is currently no guidance or regulation on risk or safety for UK residents who consider travelling abroad for treatment. Potential savings as a result of medical travel, especially evident from bariatric patients here, are noteworthy especially at a time of constrained public resources.

Our findings from NHS Trusts indicates that for those wishing to increase their private income as a result of the income cap being raised foreign private patients may be more attractive than domestic private patients.

While this particular research focused on the impact of medical tourism on the UK NHS, the findings give an indication of possible impact of medical tourism in other countries. They are likely to have particular resonance for other universal public health systems.

### Strengths and Weaknesses of the Study

While the study used the most robust data set available to measure volume of medical tourism to the UK, the International Passenger Survey, it has several weaknesses. The IPS only surveys 0.2% of travellers entering and leaving the UK. In addition, inbound figures on medical tourists do not provide information on whether these are accessing treatment in the public or the private sector. Interviews with medical tourists also suggested that not all may identify themselves as travelling for medical purposes. Moreover, costs calculated are based on published literature often drawing on small samples.

Thus, although data and analysis presented here represent the most comprehensive analysis of inbound and outbound medical tourism to date, they clearly identify the significant gap in understanding of this increasingly important phenomenon. The particular strength of the findings here lies in the mixed–methods approach. Authors undertook the first comprehensive analysis of the IPS from a medical tourism perspective. Findings were triangulated by drawing on published literature, and by analysis of interviews with 77 UK medical tourists. Similarly, the cost estimates were developed based on results from interviews, costs reported in the published literature, the IPS data set and freedom of information requests to 28 hospital foundation trusts. Hence, each finding has carefully been considered and based on more than one data source.

### Directions for Future Research

The impact of medical tourism warrants better monitoring. Findings demonstrate impact in terms of possible costs and benefits and the highly dynamic nature of the phenomenon means that the absolute numbers presented here could grow rapidly. Only continuous monitoring will allow better understanding and informed policy-making to ensure patient safety.

Estimates of cost presented here mark a first step based on the limited data available. To better understand costs and potential savings of medical tourism requires not only better data on volume of travel but also on the differences in long-term health outcomes between patients who travelled and those having received treatment at home. Further research of comparative outcomes is needed.

This research does not explore the ethical dimensions that are involved in many of the considerations relating to medical tourism, including why patients opt to receive care outside of the UK. While data here represents the economic costs of complications experienced by patients these obviously will have to be considered alongside considerations of patient safety.

### Conclusions

UK medical tourism is a growing phenomenon. To fully understand its implications and guide policy on issues such as NHS global activities and patient safety will require investment in further research and monitoring. Despite existing data limitations it is evident that UK medical tourism is dynamic and changing. Findings indicate costs arise where patients travel abroad and return with complications. Analysis also indicates possible savings in the case of specific procedures especially in future health care and social costs averted. Inbound medical tourists offer potentially high income to NHS hospitals. Results of this research may also be indicative of the impact of medical tourism in other public health systems.

### Disclaimer

The views and opinions expressed therein are those of the authors and do not necessarily reflect those of the HS&DR Programme, NIHR, NHS or the Department of Health.

## Supporting Information

Annex S1 **Cost calculations.**
(DOCX)

Annex S2 **Responses to FOI requests to 28 Foundation Trust Hospitals.**
(DOCX)
