# Supplementary material for: Medical Tourism: A Cost or Benefit to the NHS?
Source: PLoS One. 2013 Oct 24;8(10):e70406. doi: 10.1371/journal.pone.0070406 (PMC3812100; doi:10.1371/journal.pone.0070406)
Supplement: Annex S1 — Cost calculations. (DOCX) [file pone.0070406.s001.docx]

**Annex 1 Cost calculations**

**Fertility**

We drew on ONS data of multiple births in the UK for the latest available year. In 2010, 11,053 women gave birth to twins, 169 to triplets and six to quads and above, in England and Wales. A total of 11,228 multiple births.

We further assumed that the cohort studied by McKelvey et al. was slightly swayed towards medical tourists as it was based in an urban population in central London. Therefore, we assumed 20% of multiple births (a total of 2,246 women giving birth to multiples) were a result of fertility treatment received abroad. This would also be supported by our analysis of data from the IPS, where a comparatively larger number of women between (ages 24 to 45) travel abroad to access treatment.

Women with multiple pregnancies require increased and more complex monitoring, sometimes not available in routine NHS setting. Women with multiple pregnancies also require a greater number of antenatal visits. However, the exact needs are highly variable. We therefore drew on an earlier study by the Cost of Multiple Births Study Group, which calculated the expense of multiple births versus singleton pregnancy in 2006 and found that a birth of singleton was £3,313; twins £9,122; and triplet £32,354 ([1](#_ENREF_1)). We estimated that these costs had increased by 3% per year between 2006 and 2010 and then calculated the additional cost of a twin or triplet pregnancy over singleton birth (as set out in the Table 1 below) to estimate the costs through multiple births resulting from fertility travel in 2010 to be £15.5 million.

**Table 1: Annual cost of multiple births resulting from cross-border reproductive travel**

|  | **Number of births** | **Result of MT** | **Additional cost** | **Cost as result of MT** |
| --- | --- | --- | --- | --- |
| **Twins** | 11,053 | 2210.6 | 6506 | 14,382,163 |
| **Triplets** | 169 | 33.8 | 32526 | 1,099,378 |
| **Total GBP** |  |  |  | **15,481,542** |

**Cosmetic**

For our analysis we estimated, based on the IPS data of age and gender of outbound travellers that 30% (18,300) of patients travelled for cosmetic surgery. We triangulated this with the ([2](#_ENREF_2)) survey conducted by *Which?* magazine that found 28% of medical tourists travelled for cosmetic procedures, recent survey findings that 95% of all people considering cosmetic surgery would travel abroad for these ([3](#_ENREF_3)), and the survey results by medical doctors reported above. This makes it likely that these estimates are at the lower end of the spectrum.

We estimated, based on our interview findings and the survey results reported by Jeevan et al., that 10% of those who travel abroad for cosmetic treatment will require some form of aftercare, even if this is limited to dealing with an infection or removing stitching. However, drawing on Miyagi et al. we assumed a great variation in complications, with some (such as infections or the removal of stitches) being resolved at GP surgery level, for example through one consultation and a course of antibiotics. Based on our interview sample and cases reported in the literature, we assumed that 20% of complications of those travelling for cosmetic surgery would require a visit to a tertiary facility, either as an emergency or through referral. For this we used a blended figure of the four higher cost cases described by Miyagi et al. We assumed that other complications would be resolved at much lower cost (for this we used a blended cost of the two lowest costs described in Miyagi’s paper), likely to only require one GP visit to remove stitches and more complex cases dealt with at primary care level.

Based on these assumptions and drawing on the mean cost provided by Miyagi we calculated that for 2010, 1,890 patients would seek follow-up care (some major, some minor) from the NHS, at a cost of £8.2 million, as per calculation set out in the Table 2 below.

**Table 2: Annual cost of complications by returning cosmetic tourists**


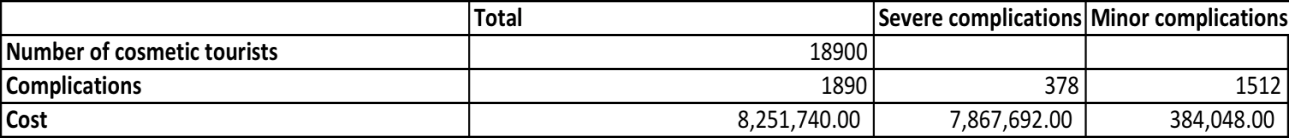


**Bariatric**

A recent study by the Office of Health Economics ([4](#_ENREF_4)) focused specifically on the cost-effectiveness of bariatric surgery in the UK. The authors estimated that between 11,000 and 140,000 people would have qualified for bariatric in 2009/10 but only 3,607 were prescribed on the NHS. The range of 11,000 to 140,000 patients is based on the assumption that between 1 and 25% of patients eligible for bariatric surgery will choose to undergo this. The authors then calculate savings to the UK economy by deducting the cost of the surgical intervention from workdays gained, and savings in social benefits, including unemployment, disability and housing benefit. Based on this range, additional income is estimated at £46 to £578 million per year, while the savings in benefit payments are estimated in the range £10 and £151 million. These calculations explicitly exclude any cost of complications or follow-up care. Further research is currently underway to examine the long-term effects of obesity surgery, specifically the SurgiCal Obesity Treatment Study (SCOTS) at the University of Glasgow.

For our sample, we used the National Tariff costs for a gastric band and gastric bypass obtained through a Freedom of Information request to calculate the cost saving from patients we interviewed who had opted to travel abroad to have bariatric surgery. The actual cost of procedures saved by the NHS was a total of £58,816 for the thirteen patients interviewed. If we use the model employed by the OHE, the total savings and additional income would rise to £112,506.

1. Ledger WL, Anumba D, Marlow N, Thomas CM, Wilson ECF, Group TCoMBS. Fertility and assisted reproduction: The costs to the NHS of multiple births after IVF treatment in the UK. BJOG: An International Journal of Obstetrics & Gynaecology. 2006;113(1):21-5.

2. Which? We expose medical tourism pitfalls: Do your homework before you go, urges Which? Which? [Internet]. 2008 1/11/2011. Available from: <http://www.which.co.uk/news/2008/03/we-expose-medical-tourism-pitfalls-135137/>.

3. Nassab R, Hamnett N, Nelson K, Kaur S, Greensill B, Dhital S, et al. Cosmetic tourism: public opinion and analysis of information and content available on the Internet. Aesthetic Surgery Journal. 2010 May;30(3):465-9. PubMed PMID: 20601579. English.

4. O'Neil P. Shedding the Pounds: Obesity Management, NICE Guidance and Bariatric Surgery in England. London: Office of Health Economics; 2010.
