# Supplementary material for: Medical Tourism: A Cost or Benefit to the NHS?
Source: PLoS One. 2013 Oct 24;8(10):e70406. doi: 10.1371/journal.pone.0070406 (PMC3812100; doi:10.1371/journal.pone.0070406)
Supplement: Annex S2 — Responses to FOI requests to 28 Foundation Trust Hospitals. (DOCX) [file pone.0070406.s002.docx]

**Annex 2**

**Responses to FOI requests to 28 Foundation Trust Hospitals**
